# Epigenetic Variation Analysis Leads to Biomarker Discovery in Gastric Adenocarcinoma

**DOI:** 10.3389/fgene.2020.551787

**Published:** 2020-12-08

**Authors:** Yan Zhang, Dianjing Guo

**Affiliations:** State Key Laboratory of Agrobiotechnology, School of Life Sciences, The Chinese University of Hong Kong, Shatin, China

**Keywords:** gastric cancer, epigenetics, histone modification, DNA methylation, biomarker

## Abstract

As one of the most common malignant tumors worldwide, gastric adenocarcinoma (GC) and its prognosis are still poorly understood. Various genetic and epigenetic factors have been indicated in GC carcinogenesis. However, a comprehensive and in-depth investigation of epigenetic alteration in gastric cancer is still missing. In this study, we systematically investigated some key epigenetic features in GC, including DNA methylation and five core histone modifications. Data from The Cancer Genome Atlas Program and other studies (Gene Expression Omnibus) were collected, analyzed, and validated with multivariate statistical analysis methods. The landscape of epi-modifications in gastric cancer was described. Chromatin state transition analysis showed a histone marker shift in gastric cancer genome by employing a Hidden-Markov-Model based approach, indicated that histone marks tend to label different sets of genes in GC compared to control. An additive effect of these epigenetic marks was observed by integrated analysis with gene expression data, suggesting epigenetic modifications may cooperatively regulate gene expression. However, the effect of DNA methylation was found more significant without the presence of the five histone modifications in our study. By constructing a PPI network, key genes to distinguish GC from normal samples were identified, and distinct patterns of oncogenic pathways in GC were revealed. Some of these genes can also serve as potential biomarkers to classify various GC molecular subtypes. Our results provide important insights into the epigenetic regulation in gastric cancer and other cancers in general. This study describes the aberrant epigenetic variation pattern in GC and provides potential direction for epigenetic biomarker discovery.

## Introduction

Gastric adenocarcinoma (GC) is one of the most common malignancies worldwide (Bray et al., 2018). Most GC patients with symptomatic tumors are diagnosed at an advanced stage (Leung et al., 2008), making GC the leading cause of death (Bray et al., 2018). Genetic and epigenetic alterations are key features acquired by cancer cells to increase fitness and drive its progression through tumor evolution (Fiziev et al., 2017). Epigenetic modifications, e.g., histone modifications and DNA methylation, have been indicated to play a considerable role in GC carcinogenesis (Klutstein et al., 2016). However, a genome-wide landscape of multiple histone marks, DNA methylation, and especially the combinatorial chromatin state in cancer progression remain largely uncharacterized, partly attributed to the lack of multiple-omics data.

As vital features of cancer, genetic, and epigenetic alterations lead to aberrant gene functions, changes in gene expression and genome stability (Jones, 2014). In contrast to genetic lesions, epigenetic changes in chromatin are biochemically reversible and involve changes in structure and function through epigenetic modifications (Allis and Jenuwein, 2016). Accumulating evidence has suggested that global levels of histone modifications are associated with clinical outcomes and progression of different cancer types (Varier and Timmers, 2011). For example, low cellular levels of H3K4me2, H3K9me2, or H3K18ac are each significant and independent predictor of poor survival in pancreatic adenocarcinoma (Manuyakorn et al., 2010); reduced H3K4me3 may have therapeutic benefit in the treatment of PI3K-activated cancers by applying chemical inhibition of the histone methyltransferase MLL1 (Spangle et al., 2016). Recent research showed that human cancer cells harbor global epigenetic abnormalities, and these genetic and epigenetic factors interact at all stages of cancer development to promote cancer progression. Previous reports suggested that infection with *Helicobacter pylori* (*H. pylori*) or Epstein-Barr virus (EBV), pathogens that play an important role in GC development, was related to increased levels of abnormal DNA methylation in GC (Calcagno et al., 2013). Many studies have also indicated that aberrant DNA methylation is not just a feature of end-stage malignancy, but also an early and driver event in gastric pathogenesis (Zeng et al., 2017; Takeshima and Ushijima, 2019). The overwhelming evidence in gastric cancer suggests that both DNA methylation and histone modification alterations co-occur, making it somewhat challenging to discern their contributions to gastric carcinogenesis. Parallel studies measuring both DNA methylation and histone modifications would be hugely valuable but might be technologically complex to achieve.

With the development of high-throughput chromatin immunoprecipitation sequencing technology (Strahl and Allis, 2000), comprehensive profiling of various epigenetic marks has now become available. Some researchers reported that H3K4me1, H3K4me3, H3K27ac, and H3K36me3 were tightly associated with active transcription (Benevolenskaya, 2007; Creyghton et al., 2010), whereas H3K27me3 was correlated with repressive loci (Barski et al., 2007). Commonly, DNA methylation is mostly associated with gene silencing (Kazmi et al., 2018). Here we systematically investigated the five core histone modification marks (H3K4me1, H3K4me3, H3K27ac, H3K27me3, H3K36me3) and DNA methylation pattern in GC samples. A comparative analysis was conducted between tumor and normal samples in this research to reveal genome-wide distinct patterns of epigenetic modifications in GC, particularly in the promoter regions. Through integrative analysis of different epigenomic and transcriptomic data, we revealed distinct patterns of oncogenic pathway activation and provided novel insights into GC subtype-specific therapeutic opportunities.

## Materials and Methods

### Epigenetic and Transcriptomic Data Sets of Gastric Cancer

All epigenetic modification data and gene expression data were collected from the primary sample research of the same patient cohort (Ooi et al., 2016), including 19 primary GCs and 19 matched normal gastric tissues (see details in Supplementary Table S1). “Normal” (i.e., non-malignant) samples used in this study were those collected from the stomach from sites distant from the tumor and without obvious evidence of tumor or intestinal metaplasia/dysplasia at the time of surgical evaluation. Tumor samples were confirmed to contain > 40% tumor cells by cryosectioning. More than 60% of the tumor were Stage 3 or above (AJCC 7th edition) (Ooi et al., 2016). The five histone modifications investigated in this study include H3K4me1, H3K4me3, H3K27ac, H3K27me3, and H3K36me3. Data generated from tumor and non-tumor adjacent tissues and input libraries were obtained from the Gene Expression Omnibus (GEO) database (GSE51776) (Muratani et al., 2014). DNA methylation data from GEO (GSE85464) (Ooi et al., 2016) were generated by Illumina HumanMethylation450 BeadChip, measuring DNA methylation levels of 485,512 CpG sites in the human genome. Gene expression data were generated from GEO (GSE85465) (Ooi et al., 2016).

### Reads Mapping

Reads generated from NanoChIP-seq were mapped to the human reference genome (hg19) by the Bowtie2 program (Langmead and Salzberg, 2012). Aligned reads were processed using samtools to remove PCR duplicates (Li et al., 2009), and read lengths were extended from 101 to 150 bp using MethylQA (Li et al., 2015). RNA-seq reads were mapped to hg19 by Tophat2 program using default parameters (Kim et al., 2013). The Cufflinks program was applied to assemble the mapped RNA-seq reads with default parameters and calculated the FPKM value (Fragments Per Kilobase of exon model per Million mapped fragments) (Trapnell et al., 2010).

### Detection of Differential Histone Modifications Regions

DiffReps program was used by default parameters for a quantitative comparison of all the five histone modification levels between tumor and non-tumor adjacent tissues (adjusted *P* < 0.05) (Shen et al., 2013). The read densities of the NanoChIP-seq library were corrected against the corresponding input library.

### Detection of Differential DNA Methylation Regions

Differential methylated regions (DMRs) were obtained by the DMRcate program using default parameters (Peters et al., 2015). The candidate DMRs with an FDR < 0.05 (Benjamini-Hochberg) were identified as differentially methylated regions. Differentially methylated positions (DMPs) were identified by the minfi program (Aryee et al., 2014), which employed the *F*-test to compare CpGs in the tumor and control samples, and the CpGs with *q*-value < 0.05 were identified as differentially methylated positions.

### Identification of Genomic Feature With Epigenetic Modifications

To investigate the potential co-localization relationships between epigenetic alterations, genome-wide overlap analysis was performed for each pair of epigenetic alterations by bedtools intersect, and significance tests were performed using the bedtools fisher (Quinlan and Hall, 2010). The differential epigenetic alterations were mapped to various genomic features, including promoter (< 1 kb), promoter (1–2 kb), UTR5, first-exon, other-exon, first-intron, other-intron, UTR3, downstream (of gene end), and distal intergenic for the hg19 genome. Promoter regions were defined as region 2 kb up- and down-stream of the transcription start sites (TSSs) of genes. The genomic feature with altered epigenetic modifications was identified by ChIPseeker (Yu et al., 2015).

### Functional Enrichment Analysis

The R package clusterProfiler and gene annotation tool Metascape were adopted to uncover the functional enrichment (Yu et al., 2012; Zhou et al., 2019).

### Chromatin States Analysis

ChromHMM enables the learning of chromatin states, annotates their occurrences across the genome by automatically computing state enrichments for external annotations (Ernst and Kellis, 2012). The narrow peak files of histone modifications obtained from MACS2 were used as input data using a *P*-value cutoff of 1e-4 (Zhang et al., 2008). The analysis was conducted by 19 states based on the theory from previous research (Fiziev et al., 2017). The chromatin states were annotated according to the functional annotations of the human genome (Ernst and Kellis, 2010).

### Chromatin State Transition of the Tumor and Normal Samples

Chromatin state transition probability between normal and tumor cells was calculated based on the method described before (Fiziev et al., 2017). The 200 bp bins were counted based on the segment’s information supplied by each of tumor and normal samples. Then, we intersected the bins occupied by 19 states annotations of the tumor and normal samples, respectively. To calculate the raw enrichment score, the number of intersected bins (*Num*_*observed*_) were divided by the expected number of such bins (*Num*_*expected*_) assuming a null model that the chromatin states of tumor cells and chromatin states of normal cells were independent.

$$R\,E\,S\,\left(R\,a\,w\,E\,n\,r\,i\,c\,h\,m\,e\,n\,t\,S\,c\,o\,r\,e\right)=\frac{N\,u\,m_{o\,b\,s\,e\,r\,v\,e\,d}}{N\,u\,m_{e\,x\,p\,e\,c\,t\,e\,d}}$$

Further, to normalize the enrichment score, we divided the enrichment score of transitioning from state *i* in normal samples to state *j* in tumor samples (*RES*_*N_i T_j*_) by the enrichment score of transitioning from state *j* in normal samples to state *i* in tumor samples (*RES*_*N_j T_i*_).

$$N\,E\,S_{N_{i}\,T_{j}}\,\left(N\,o\,r\,m\,a\,l\,i\,z\,e\,d\,E\,n\,r\,i\,c\,h\,m\,e\,n\,t\,S\,c\,o\,r\,e\right)=\frac{R\,E\,S_{N_{i}\,T_{j}}}{R\,E\,S_{N_{j}\,T_{i}}}$$

### The Analysis of Epigenetic Regulation of Gene Expression

To examine the association between epigenetic alterations and gene expression patterns, genes involved in epigenetic modifications were grouped according to the characteristics of the modifications. The genes were first divided into four distinct groups based on the number of co-localizations of various epigenetic modification alterations in their promoter regions. Genes with a single type of epigenetic modification, genes with two types of epigenetic modification, genes with three types of epigenetic modification, and genes with no less than four types of epigenetic modification alterations. The four groups of genes were then further classified into three different subgroups of active, repressed, or poised genes based on the effect of epigenetic modification alterations on the transcription of the corresponding genes, respectively. As described above, H3K4me1, H3K4me3, H3K27ac, and H3K36me3 serve as active signals, whereas H3K27me3 and DNA methylation are repressive signals. The active subgroups included genes regulated by up-regulated active and down-regulated repressive signals, whereas the repressive subgroups contained genes regulated by down-regulated active signals and up-regulated repressive signals, and the poised subgroups contained genes with conflicting epigenetic signals that were either up-regulated active and up-regulated repressive signals, or down-regulated active and down-regulated repressive signals. We calculated the average log2 FPKM fold change for genes between tumor and normal samples in each subgroup. Then, for the following hub gene screening, overexpressed genes whose log2 FPKM fold change > 1 in the active subgroups and underexpressed genes whose log2 FPKM fold change < −1 in the repressive subgroups were retained (paired *t*-test, *P*-value < 0.05) (Ernst et al., 2017).

### Construction of Protein-Protein Interaction (PPI) Network for Hub Genes

To obtain the epigenetically regulated GC oncogenes, we searched the oncogene database^1^ (Pletscher-Frankild et al., 2015). String (Szklarczyk et al., 2018) was used to construct the PPI network, and 293 genes resulted. The hub genes were discerned by Cytoscape (Smoot et al., 2010). To filter out the hub genes from genes involved in the PPI network, we focused on genes that could be the potential targets of the epigenetic change. We first obtained overexpressed genes in the active subgroups and underexpressed genes in the repressive subgroups based on the analysis results. Genes with the “closeness” parameter over 130 were then screened for the next step. Because the number of overexpressed genes was far more than underexpressed genes, we sampled all underexpressed genes and the top 30% of overexpressed genes based on the sort of “Degree” parameter. At last, we obtained 53 genes for further analysis.

Reactome pathway enrichment was performed by the ClueGO function of Cytoscape, and the association between different pathways was calculated according to the kappa score (Croft et al., 2013), which is used to define the term-term interrelations (edges) and functional groups based on shared genes between the terms.

### Consensus Clustering

The hierarchical clustering of RNA-seq data was conducted using the ward.D2 agglomeration method and Euclidean distance. All 32 normal samples and corresponding tumorous samples were screened. The gene expression data of breast invasive carcinoma (BRCA), colon adenocarcinoma (COAD), liver hepatocellular carcinoma (LIHC), and thyroid cancer (THCA) were collected from The Cancer Genome Atlas (TCGA).

### Cox Regression Model

The Cox regression model was used to evaluate the association between survival and the expression level of each hub gene (Cox, 1972). Genes that were significantly correlated with survival (*P* < 0.05) were identified as members of the gene signature. Furthermore, we assigned each patient a risk score according to a linear combination of the gene expression values weighted by the regression coefficients from the univariate Cox regression model. The risk score for each patient was calculated as follows:

$$R\,i\,s\,k\,\_\,s\,c\,o\,r\,e=\sum_{i=1}^{n}\beta_{i}\times E\,x\,p_{s\,i\,g\,n\,a\,t\,u\,r\,e\,\left(i\right)}$$

where β_*i*_ is the Cox regression coefficient of gene *i*, and *n* is the number of genes significantly associated with survival. All patients were thus divided into high-risk and low-risk groups using the median risk score as the cutoff. The Kaplan-Meier method was further used to estimate the overall survival time for the four molecular subtypes. The differences in the survival times were analyzed by the log rank test.

## Results

### Epigenetic Modification Landscape in Gastric Cancer

In this study, the differential epigenetic modified regions (DEMRs) by five core histone modifications and DNA-methylation in GC were identified (Table 1). In total, 8,424 DMRs were identified in GC (FDR < 0.05). A detailed investigation of the differential methylation positions (DMPs) revealed 90,468 differentially methylated CpGs sites, among which 18,460 were found within the CpG islands (CGIs) (*q* < 0.05, *F*-test, FDR-corrected). Most of the DMPs were hypomethylation (87%, *P* < 2e-16, Wilcoxon rank-sum test) (Supplementary Figures S1A,B) associated with non-CGIs (*P* < 2.2e-16, Chi-square test, Yates’ continuity corrected). Hypermethylation was found to be mostly associated with CGIs (*P* < 2.2e-16, Chi-square test, Yates’ continuity corrected) (Supplementary Figures S1C,D).

**TABLE 1:** Counts of differentially epigenetic modified regions (DEMRs) for six types of epigenetic modifications in GC.

Epigenetic modifications	DEMRs	Up/down
H3K4me1	2,744	1,617/1,127
H3K4me3	3,559	1,701/1,858
H3K27ac	7,557	4,856/2,701
H3K27me3	1,392	978/414
H3K36me3	4,453	2,690/1,763
mC	8,424	1,156/7,268

**FIGURE 1:**
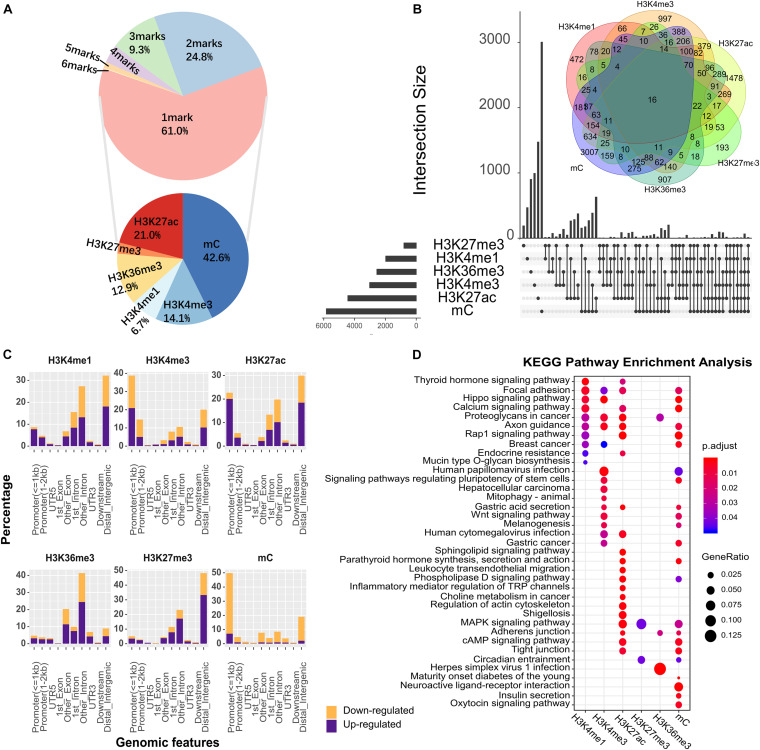
The epigenetic alteration for different genomic features in GC. **(A)** The proportion of genomic regions occupied by different numbers of epigenetic alterations. In the upper pie chart, 61% of genomic region were occupied by 1 epigenetic mark, and the remaining regions were co-occupied by varying numbers of epigenetic marks. Base on the genomic region occupied by 1 mark, the lower pie chart showed the proportion of genomic regions occupied by different types of epigenetic marks. **(B)** Overview of co-localization for each epigenetic modification. The intersection area indicates the count of co-altered epigenetic modifications, corresponding to the number in the Venn diagram. Different types of epigenetic marks altered together were connected by black lines. **(C)** The percentage of genomic features with altered epigenetic modifications. Upregulated and downregulated epigenetic modifications were colored in purple and orange, respectively. **(D)** KEGG enrichment analysis for all DHMRs. The *X*-axis denotes different types of epigenetic modification.

Epigenetic modifications show a specific degree of redundancy, and certain epigenetic modifications may work in a combinatorial manner (Wang et al., 2008). We found that ∼49.7% of the genes in the genome were associated with at least one type of epigenetic change, and 24.8% of the genomic regions were marked by two co-localized epigenetic modifications (Figure 1A). H3K27ac/mC (Fisher’s two-tailed *P* = 6.5598e-292), H3K4me3/mC (Fisher’s two-tailed *P* = 3.0205e-273), and H3K4me3/H3K27ac (Fisher’s two-tailed *P* = 0) were commonly found pairs that were highly significant (Figure 1B), and the most frequent triplet marks were H3K4me3/H3K27ac/mC (Fisher’s two-tailed *P* = 4.8021e-150) and H3K4me1/H3K27ac/mC (Fisher’s two-tailed *P* = 7.8464e-17). Among genes modified by the six types of epigenetic modifications in Figure 1B, *AOC1* was an oncogene in human gastric cancer that activates the AKT signaling pathway (Xu et al., 2020). *MYC* has been described as a key factor in several human carcinogenic processes (Calcagno et al., 2008). The overexpression of *PRKCI* was associated with poor outcomes in patients with gastric and other cancers (Hashimoto et al., 2019). Upregulation of *BCAT1* was associated with poor prognosis in numerous types of tumors and its high expression significantly worsen overall survival in gastric tumors (Xu et al., 2018). To systematically investigate the distribution of epigenetic alterations in different genomic regions, the genome was portioned into 10 regions (Figure 1C). UTR5, UTR3, first-exon and downstream were the least favored regions by altered epigenetic marks. Promoters were the main targets of DNA methylation (54.45%) and H3K4me3 modification (53.41%). Altered H3K36me3 was mostly mapped to the coding sequence and introns (71.79%).

To explore the biological significance of genes regulated by epigenetic alterations, we next performed the Kyoto Encyclopedia of Genes and Genomes (KEGG) pathway enrichment analysis for these genes (Figure 1D). Among the top enriched pathways, some were shared by different epigenetic alterations. For example, “Gastric Cancer” and “Wnt signaling pathway” etc. On the other hand, a fair number of pathways were associated with only one specific epigenetic alteration, e.g., “Herpes simplex virus 1 infection,” which is involved in *Helicobacter pylori* infection (Tsamakidis et al., 2005). Overall, these observations suggested that epigenetic regulation plays an essential role in cancer-associated biological processes.

### The Chromatin States Shift in Gastric Cancer

Chromatin states and their genomic occurrences provide a systematic annotation of DNA elements and regulatory regions and can be used to interpret genome-wide association of epigenetic modification and gene expression in cancer (Filion et al., 2010; Ernst et al., 2011).

**FIGURE 2:**
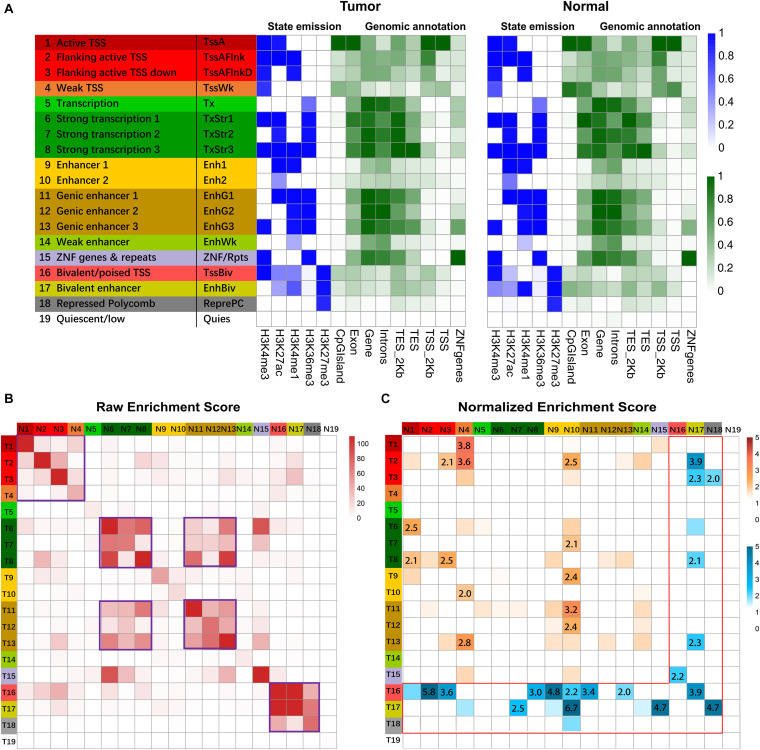
Chromatin state analysis. **(A)** ChIP signals matrix showed the histone modification profiles of tumor samples and normal samples for the 19 states inferred by the ChromHMM algorithm. First column gave state number and candidate state description, and second column gave the state abbreviations. In the heatmap of the emission parameter, each row corresponded to a different state, and each column corresponded to a different histone mark. The darker blue color corresponded to a higher frequency of occurrence of the mark in the state on the scale from 0 (white) to 1 (blue). The heat map of Genomic annotations displays enrichment for various external genomic annotations. A darker green color corresponded to a higher enrichment on the genomic feature. Overlap of different genomic features (CpG island, Exon, Gene, Intron, TES_2kb, TES, TSS_2kb, TSS, ZNF genes) with chromatin state called in tumor and normal cells. TES indicated transcription end site, and TES_2kb indicated regions within 2kb of the TES. TSS indicated transcription start site, and TSS_2kb indicated regions within 2kb of TSS. **(B)** Heatmap showed the raw enrichment score of state transitions between normal and tumor samples. The color bars on the left and top corresponded to the color bar of state description in **(A)**. “T” on left color bar indicated tumor sample, and “N” on the top color bar indicated normal samples. The frequent state transitions were highlighted with purple frame. **(C)** Heatmap showed normalized enrichment score of transitions of chromatin states from normal to GC samples. The color intensities range from white (relative enrichment < 1) to orange and blue (relative enrichment > 1). The normalized enrichment score of more than two were shown. The region with non-repressive states was labeled with orange color, and the region with the repressive state was labeled with a blue color with red frame.

A combinatorial chromatin state transition analysis was conducted for GC vs. normal samples. A final model with 19 states was adopted for further downstream analysis (Figure 2A). By triangulating the defined chromatin states with known genome organization features, we grouped the 19 chromatin states according to the following putative annotations: promoter regions (state 1–4), transcribed (state 5–8), enhancers (state 9–14), Zinc finger genes (state 15), bivalent promoter regions (state 16), bivalent or weak enhancer (state 17), polycomb repressed (state 18), and quiescent (state 19).

Pairwise state transitions between normal and GC samples were investigated (Figure 2B), and frequent transitions were found between closely located genomic regions, e.g., promoter regions (state 1–4). Pearson’s chi-square test for independence further supported the above findings (Supplementary Figure S2). To further understand the state transitions, we normalized the enrichment of state transitions between normal and GC samples with respect to the same pair with opposite directions. Some predominant transitions between states were identified. For example, the frequency of state transition from weak TSS (state 4) to active TSS (state 1) is 3.8 times more compared with active TSS to weak TSS transition (Figure 2C). Although frequent transitions were found between specific genomic regions, the frequency of many such transitions was equal between the same pair of states from normal to GC samples. For instance, the frequency for state 6 to state 7 transition was equivalent to that for state 7 to state 6 transitions (Figure 2C).

To explore the biological significance of predominant chromatin state transition, we performed pathway enrichment analysis for genes associated with the specific pairwise transition in their promoter regions. We found that promoters harboring weak to active TSS transition in GC were preferentially mapped to several cancer-associated terms, such as “cell cycle phase transition,” “cell division,” “DNA repair” (Supplementary Figure S3A), suggesting increased cell division and accelerated cell cycle in GC. Meanwhile, sizable genes switched from Active/Flanking TSS to Bivalent/Poised TSS in GC were enriched with GO term such as “regulation of cell adhesion” and “negative regulation of cell differentiation,” consistent with the fact that many malignant tumors are dedifferentiated cells bearing little or no resemblance to the normal cells (Supplementary Figures S3B–D).

Overall, these results suggest that transition from normal to tumor phenotypes is accompanied by chromatin states transition within specific regions. In particular, our results revealed significant predominant epigenetic transitions from normal to tumor cells, indicating the crucial role of combinatorial histone modifications in GC.

### The Combined Effects of Histone Modifications and DNA Methylation on Gene Expression

Histone modifications and DNA methylation are two key factors regulating gastric carcinogenesis. However, whether these two factors function independently or coordinately in GC is still unknown. In our study, we first examined the impact of DNA methylation on gene expression and found that 61.4% of the genes exhibited a significant negative correlation (*r* = −0.436, *P* = 1.12e-156) (Supplementary Figures S4A,B). Hence, we used this group of genes for the following correlation analysis.

**FIGURE 3:**
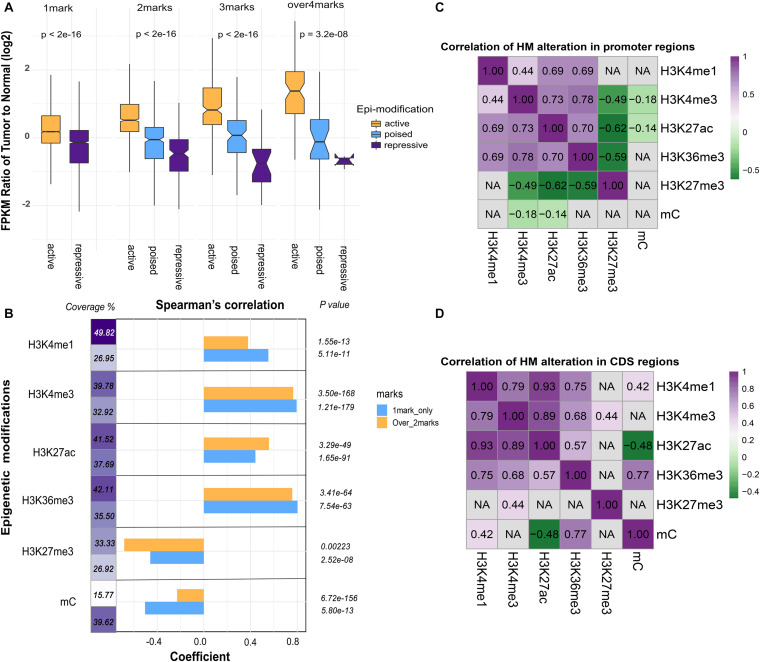
The association between epigenetic modifications and gene expression. **(A)** Additive effects of epigenetic alterations on gene expression. Genes were grouped into active subgroups (orange), poised subgroups (blue), and repressive subgroups (purple). The *X*-axis denotes the counts and patterns of epigenetic marks, and the *Y*-axis shows the log2 FPKM fold change of gene expression. **(B)** The coefficient of Spearman’s correlation was calculated between epigenetic alterations and fold change of gene expression. Promoters of genes modified by only one epigenetic mark (blue) or more than two marks (orange) were indicated. The coverage denotes the proportion of genes in each category. **(C)** Pearson’s correlation analysis of paired epigenetic alterations at the promoter and **(D)** the coding DNA sequence (CDS) (*P* < 0.05). Non-significant results were denoted with “NA.”

To assess the relationship between combined epigenetic marks and gene expression, genes were divided into four categories according to the number of differential epigenetic modifications at their promoter regions. As described above, H3K4me1, H3K4me3, H3K27ac, and H3K36me3 serve as active signals, whereas H3K27me3 and DNA methylation are repressive signals. Among the four categories, these genes were further classified into active, repressive, or poised subgroups according to the effect of epigenetic modifications. As seen in Figure 3A, the global gene expression levels in the active subgroups were increased, while those in the repressive subgroups were decreased. Furthermore, with increased number of altered epigenetic marks, the additive effect became more apparent and accumulative. Notably, with the increased number of significantly altered epigenetic modifications, the regulatory effects of multiple epigenetic marks tend to be significant and effective. Thus, these results suggested that various epigenetic modifications may function synergistically to regulate gene expression.

To further investigate the pathways regulated by the combined epigenetic alterations, we performed the KEGG pathway enrichment analysis of up- and down-regulated genes in each subgroup (Table 2). First, several cancer-associated pathways were identified in each of the subgroups. For example, in the 1-marker group, the “cell cycle” pathway was activated, and in the 2-markers group, the “gastric cancer” pathway was activated and the “apoptosis” pathway was inhibited. Thus, we obtained information on cancer-related pathways associated with epigenetic alterations, as well as how the combinations of epigenetic alterations regulate the relevant pathways (activation/repression).

**TABLE 2:** KEGG enrichment analysis of up- and down-regulated genes in each subgroup with *P*-value cutoff 0.05.

Groups	Subgroups	Term ID	Description	−log10(P)
1 Marks	Active	hsa04080	Neuroactive ligand-receptor interaction	7.13
		ko04110	Cell cycle	3.35
		ko05033	Nicotine addiction	2.78
	Repressive	ko04270	Vascular smooth muscle contraction	4.87
		hsa04911	Insulin secretion	4.72
		ko04977	Vitamin digestion and absorption	2.93
2 Marks	Active	ko04610	Complement and coagulation cascades	4.65
		hsa00350	Tyrosine metabolism	3.52
		hsa05226	gastric cancer	3.00
	Poised	ko04137	Mitophagy–animal	2.29
		ko04722	Neurotrophin signaling pathway	2.04
		hsa00515	Mannose type O-glycan biosynthesis	1.61
	repressive	ko04024 hsa04728	cAMP signaling pathway Dopaminergic synapse	3.37 3.12
		ko04210	Apoptosis	1.82
3 Marks	Active	ko05160	Hepatitis C	4.13
		ko04914	Progesterone-mediated oocyte maturation	2.40
		hsa04392	Hippo signaling pathway–multiple species	2.39
	Poised	ko04971	Gastric acid secretion	2.92
		ko05414	Dilated cardiomyopathy	2.69
		hsa05200	Pathways in cancer	2.27
	Repressive	ko04960 ko04070 ko04144	Aldosterone-regulated sodium reabsorption Phosphatidylinositol signaling system Endocytosis	3.50 2.66 1.84
Over 4 Marks	Active	hsa04146	Peroxisome	2.07
	Poised	NA	–	–
	Repressive	NA	–	–

**−log10(P) indicated the −log10 P-value for enrichment. The top three enriched terms were shown based on the sort of −log10(P). Non-significant results were denoted with “NA.”**

To compare the influence of unique and multiple epigenetic modifications at promoter regions, we grouped the differentially modified genes based on the number of epigenetic marks (one mark group and more than one mark group). Interestingly, the correlation between fold change of DNA methylation and gene expression was weak in the latter group. The effect of DNA methylation tends to be more evident when it acts alone (Figure 3B). In addition, the proportion of genes modified by DNA methylation and other histone modifications was far less than the genes altered by DNA methylation only (Figure 3B). This observation indicated that DNA methylation tends to function independently. Then, the pairwise correlation of epigenetic alterations was investigated for promoter and gene body region, respectively (Figures 3C,D). In general, relatively strong associations were maintained among the five core histone modifications at the promoter regions, whereas the correlation between DNA methylation and histone modifications was weak.

### Distinct Oncogenic Pathways Associated With Epigenetic Modifications

Systematic characterization of gastric cancer genomes has identified somatic mutations in several key signaling pathways (Kimura et al., 2004). Globally, most of the frequently mutated genes, such as *MYC*, *KARS*, *MDM2*, were also regulated by various types of epigenetic modifications, implying the interactions between genetic and epigenetic processes in tumor onset and progressions. In the critical signaling pathways of gastric cancer, the alteration of epigenetic modification of genes may result in distinctive biological consequences.

**FIGURE 4:**
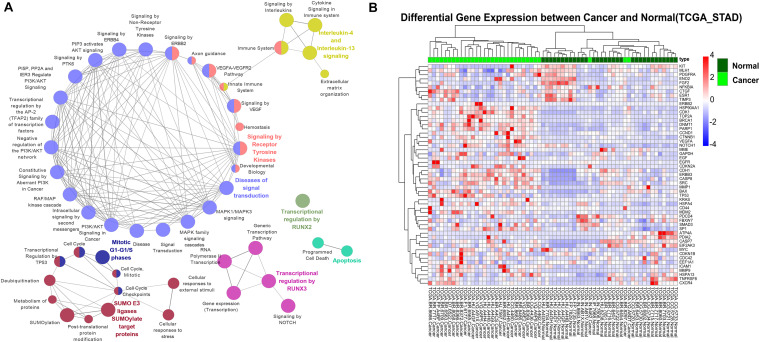
The association between oncogenic pathways and epigenetic modifications. **(A)** The Reactome pathway enrichment analysis for 53 essential hub genes. **(B)** Hierarchical clustering of 64 gastric cancer samples (32 tumor samples and 32 corresponding non-tumor adjacent samples) from TCGA using expression profiles of epigenetically regulated genes in key signaling pathways.

To explore the effect of epigenetic alteration on oncogenic pathways, we first filtered the genes with multiple epigenetic modifications in the oncogene database. A protein-protein interaction network (PPI) was then constructed to identify the vital hub genes. In total, 53 genes were identified as potential essential gastric cancer-related genes regulated by epigenetic modifications. We next performed pathway enrichment analysis for these essential genes through the Reactome pathway database search (*P* < 0.05) (Figure 4A). Among the enriched pathways, “ERBB2 signaling pathway” contains essential genes *ERBB2*, *ERBB3*, *EGF*, *EGFR*, *KRAS*, and *SRC*. *EGFR* plays a role in gastric mucosa proliferation and gastric cancer development. Overexpression of *EGFR* was found associated with poor cancer prognosis. One of the downstream components of EGFR pathways is Ras, an oncogenic GTPase that has three isoforms, including *KRAS*, *HRAS*, and *NRAS*. Mutation of *KRAS* gene has been detected in the intestinal type of gastric cancer (Molaei et al., 2018).

### Clinical Indications of Crucial Pathway Genes Modified by Epigenetic Marks

The epigenetically regulated key genes may serve as important indications in the clinical practice of GC. We found that the 53 key oncogenic pathway genes showed significant tumor-specific expression patterns in the clinical samples. Hierarchical clustering of the TCGA gastric cancer genome using these key genes resulted in a consistent separation of tumors vs. normal groups (Figure 4B and Supplementary Figure S5). Promisingly, these genes could also be used as general markers for other cancer types. For example, clear separations of tumor vs. normal samples were also achieved for human breast cancer, colon cancer, hepatocellular liver carcinoma, and thyroid cancer (Supplementary Figure S6). This result highlights the importance of these key biomarker genes in the general diagnosis of different cancer types.

**FIGURE 5:**
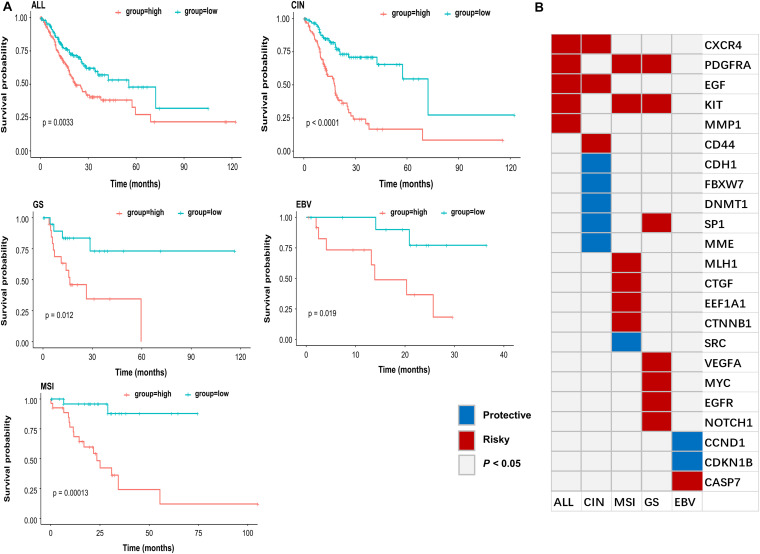
Subtype-specific progression associated genes. **(A)** Kaplan-Meier estimates of overall survival rate for all samples (ALL), CIN, GS, EBV, and MSI subtype of TCGA patient cohort according to the expression pattern of subtype-specific genes in **(B)**. Red line indicated that GC patients with high-risk scores were associated with a lower median survival rate, and green line indicated that GC patients with low-risk scores were associated with a higher median survival rate. **(B)** Subtype specific gastric cancer progression associated genes (*P* < 0.05 were shown). Red (Risky) indicated that higher gene expression associated with worse survival, and blue (Protective) indicated that higher gene expression associated with better survival.

To further explore whether these genes can be effectively used as a prognosis signature, e.g., the survival of GC patient, Cox regression analysis was performed to evaluate the effect of gene expression on the GC patient status. All gastric tumor patients of TCGA were divided into high-risk and low-risk groups based on the risk scores calculated from the formula described in the method. As shown in Figure 5A, GC patients with high-risk scores were associated with a lower median survival rate compared to those with low-risk scores. These identified key oncogenes were also found effective in various GC subtypes. Histologically, gastric tumors were classified into intestinal and diffuse types according to the Lauren’s classification, and current histopathologic systems can influence the choice of endoscopy or surgery to some extent (Sanjeevaiah et al., 2018). Besides, the TCGA has proposed a molecular classification method to divide GC into four subtypes: EBV-positive tumors, microsatellite unstable tumors (MSI), genomically stable tumors (GS), and tumors with chromosomal instability (CIN) (Network, 2014; Cristescu et al., 2015). Considering the heterogeneity of the disease and the guidance for precise treatment of individual patients, such molecular data-based classification may prove to be more clinically influential in therapeutic prediction and prediction of patient prognosis (Chia and Tan, 2016). Among the 53 genes, 23 were identified as GC-specific prognostic markers based on the four molecular subtypes (*P* < 0.05) (Figures 5A,B). The marker genes identified in this study may provide further opportunity for epigenetic targeted cancer therapy.

## Discussion

In this work, a genome-wide landscape of epigenomic variation in gastric cancer was portrayed based on reasonable sample series and rigorous statistical analysis. At the genome level, epigenetic alterations were frequently found in GC. To examine the histone modification pattern in GC, we carried out chromatin state transition analysis. Notably, the feature of non-bivalent chromatin states was rather stable. Accompanied by chromatin state transition in GC, certain combinatorial histone marks tend to label different sets of genes in the GC genome compared to control. The predominant chromatin states transition suggested that this pattern of combinatorial histone modification may functionally dysregulate gene expression in GC. Pathway enrichment analysis showed that the predominant state transition was involved in several cancer-associated GO terms, including “cell cycle,” “cell division,” “DNA repair,” “regulation of cell adhesion,” “negative regulation of cell differentiation,” and “response to wounding” etc.

DNA methylation and histone modification influence the genome function through changing chromatin architecture and stability. For the first time, we revealed that multiple epigenetic modifications might regulate gene expression synergistically, and their effects are accumulative. Interestingly, we found that the impact of DNA methylation on gene expression was more significant without the presence of histone modifications, suggesting that histone modification tends to mock the effect of DNA-methylation when both marks are present.

Epigenetically modified genes were mapped to distinct oncogenic pathways by constructing a PPI network. A series of notable pathways dysregulated by multiple epigenetic modifications were revealed by using the key genes. For example, the “SUMOylation pathway” genes were enriched, including *BRCA1, CDKN2A, DNMT1, ESR1, MDM2, NFKBIA, PARP1, TOP2A, and TP53*. SUMOs (small ubiquitin-like modifiers) are ubiquitin-like proteins that become conjugated to substrates through a pathway that is biochemically similar to ubiquitination (Poukka et al., 2000). Recently, dysregulated SUMOylation has been observed in human cancers (Kim and Baek, 2006; Eifler and Vertegaal, 2015). However, there is no study focusing on the influence of sumoylation-related genes on the risk of GC. Our study revealed that the “SUMOylation pathway” genes not only associated with GC but also regulated by epigenetic modifications. Thus, the “SUMOylation pathway” may be a potential target for epigenetic cancer therapy. Furthermore, the hierarchical clustering of the TCGA gastric cancer genome using these key genes resulted in a precise grouping of tumors from normal samples. Promisingly, these key genes are also efficient in the classification of other types of cancers, such as breast cancer, colon cancer, hepatocellular liver carcinoma, and thyroid cancer.

By evaluating the significant association between gene expression and overall survival, we identified some potential biomarkers for all gastric cancer, as well as CIN, MSI, GS, and EBV subtype, respectively (Figure 5B). For example, *EGF* and *MYC*, the well-known oncogenes in GC (Cai et al., 2019), were identified as general markers. Although some of the marker genes are commonly found in different GC subtypes, most of them were subtype specific. For instance, *CD44* showed increased resistance for chemotherapy- or radiation-induced cell death (Takaishi et al., 2009) and was previously identified as a marker gene for gastric cancer stem cells. Our study revealed that *CD44* was likely the distinct biomarker for CIN subtype. Besides, the previous study indicated that *CCND1* overexpression was associated with a more favorable prognosis and responded better to anti-estrogen therapy in breast cancer (Bieche et al., 2002). In our study, we identified *CCND1* as the potential specific biomarker for the EBV subtype. Our results suggested that subtype-specific epi-regulated biomarkers tend to associate with the overall survival of patients. Such findings may facilitate the prognosis of gastric cancer subtype in clinical practice.

## Conclusion

In summary, we first carried out a comprehensive investigation of various epigenetic alterations in GC. Through systematic profiling of six epigenetic modifications and transcriptomic analysis, we defined the chromatin state transition associated with tumorigenesis of gastric adenocarcinoma. The combined effects of multiple epi-modification marks on gene expression were then discussed. The results of the additive effect analysis of epigenetic alteration in gene expression not only explained the manner of epigenetic regulation, but also gave us information on what pathway would be affected through combined epigenetic modifications. Meanwhile, the results suggested a possible interplay among histone modification and DNA methylation. Finally, we identified a list of potential prognostic biomarker genes regulated by epigenetic modifications. Our findings will facilitate more accurate classification and diagnosis of patients with gastric cancer and hold premises for better prevention and therapy of gastric cancer as well as other cancer types in general.

## Data Availability Statement

The datasets presented in this study can be found in online repositories. The names of the repository/repositories and accession number(s) can be found in the article/Supplementary Material.

## Author Contributions

YZ and DG conceived the project. YZ designed this study, analyzed and interpreted the data, and wrote and edited the manuscript. DG reviewed and revised the manuscript. Both authors read and approved the final manuscript.

## Conflict of Interest

The authors declare that the research was conducted in the absence of any commercial or financial relationships that could be construed as a potential conflict of interest.

## Footnotes

1 https://diseases.jensenlab.org/Search
