# Supplementary material for: Epigenetic Variation Analysis Leads to Biomarker Discovery in Gastric Adenocarcinoma
Source: Front Genet. 2020 Dec 8;11:551787. doi: 10.3389/fgene.2020.551787 (PMC7753064; doi:10.3389/fgene.2020.551787)
Supplement: Supplementary file 1 [file Data_Sheet_1.docx]

# Epigenetic variation analysis leads to biomarker discovery in gastric adenocarcinoma

Yan Zhang^1^, Dianjing Guo^1*^

**Supplementary Information**

**Figure S1 Gastric cancer and normal samples methylomes**

**Figure S2 Pearson’s residual of Chi-squared test of chromatin state transition**

**Figure S3 Chromatin state transition between normal and tumor cells**

**Figure S4. Methylation-expression correlation of gastric cancer**

**Figure S5 Hierarchical clustering of independent gastric cancer samples cohorts**

**Figure S6 Hierarchical clustering of four other different types of cancer**

**Table S1 Datasets used in this study**


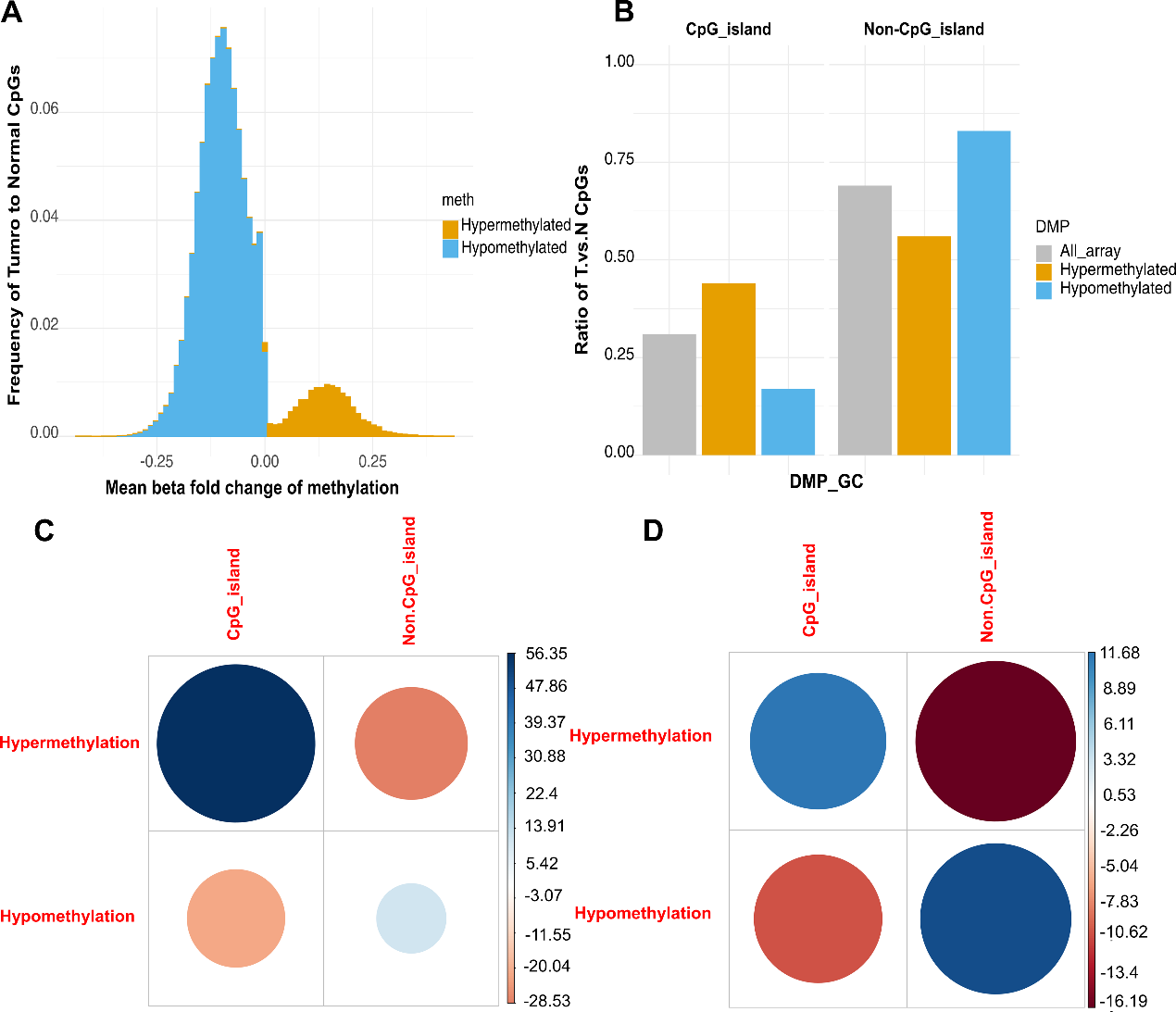


**Figure S1. Gastric cancer and normal samples methylomes. (A)** Normalized histogram of differentially methylated CpGs. hypermethylated and hypomethylated CpG sites in GC were labeled in orange and blue respectively. **(B)** Frequencies of hypermethylated (orange bars) and hypomethylated (blue bars) CpGs in CGI and non-CGI in the dataset. **(C)** Pearson residuals of Chi-square test to evaluate the association of hyper or hypo DNA methylation and CGIs or non-CGI for the GEO dataset and **(D)** TCGA gastric cancer dataset. Positive residuals (blue) specified a positive association between the corresponding row and column variables, and negative residuals (red) specified no association between the corresponding row and column variables. For a given cell, the size of the circle is proportional to the amount of the cell contribution.


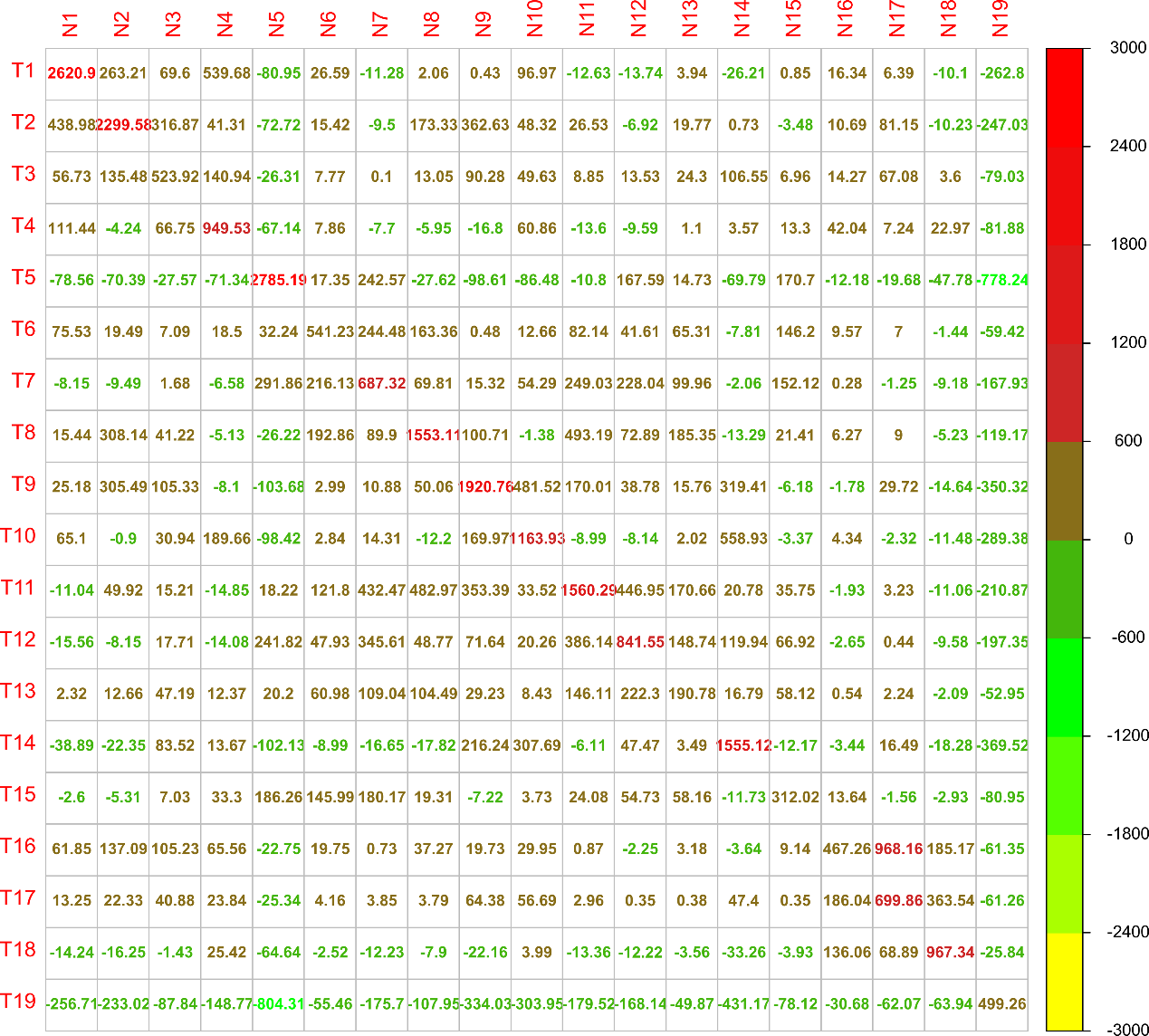


**Figure S2. Pearson’s residual of Chi-squared test for chromatin state transition between normal (N) and tumor (T).** Pearson’s residual of the positive association between the corresponding state of the tumor and normal was more than zero, indicated with brown to red color, which means the state of column and row were correlated. Conversely, if the residual was negative, the state of column and row were not correlated. This result further supported the raw enrichment score analysis.


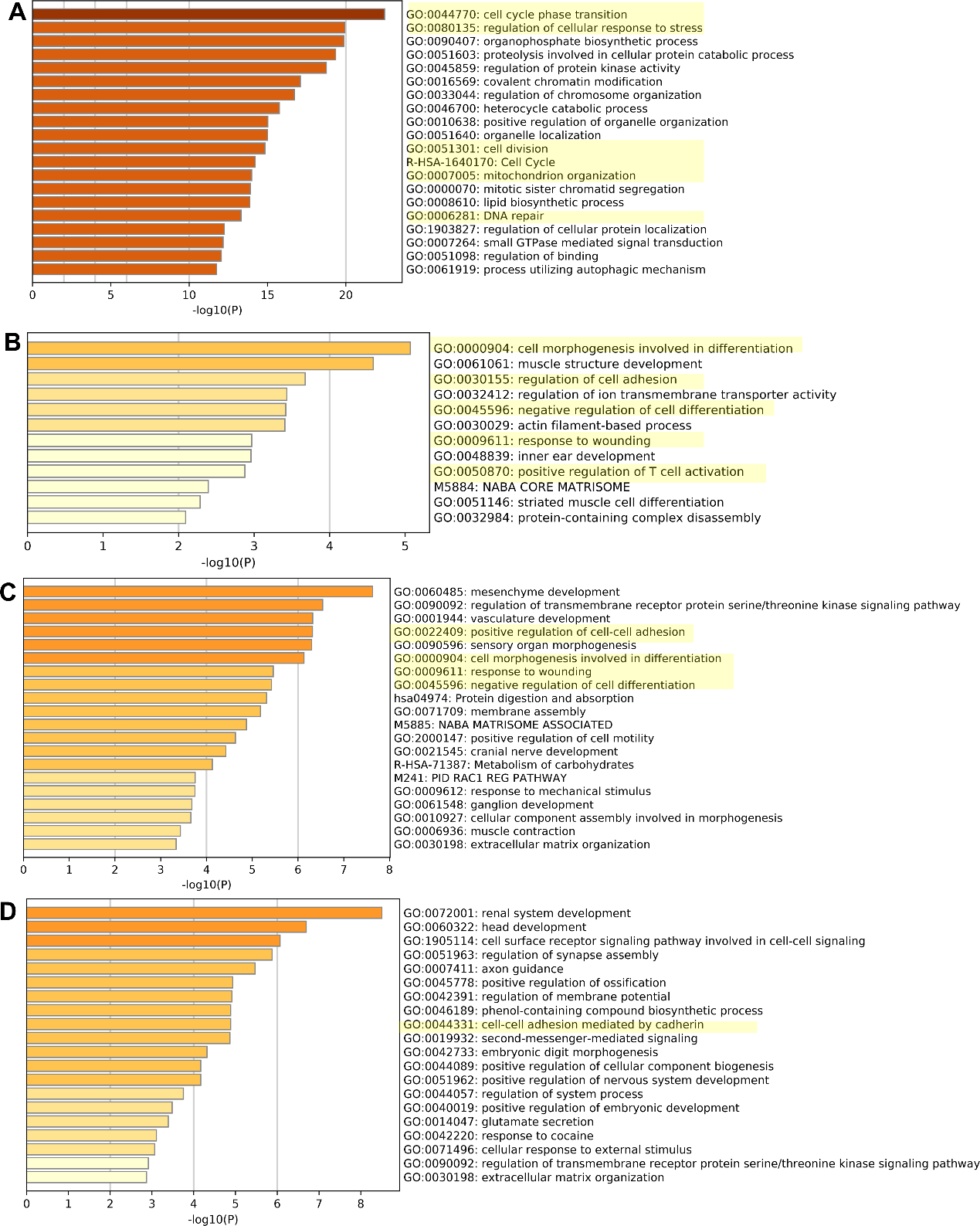


**Figure S3. Chromatin state transition between normal and tumor cells.** (**A**) GO and KEGG enrichment analysis of genes whose chromatin state transferred from “Weak TSS” (state 4) of normal cells to “Active TSS” (state 1) of tumor cells. The X-axis indicated the -log10 p-value for enrichment. (**B**) GO and KEGG enrichment analysis of genes whose chromatin state transferred from “Active TSS” (state 1) of normal cells to “Bivalent/poised TSS” (state 16) of tumor cells. (**C**) GO and KEGG enrichment analysis of genes whose chromatin state transferred from “Flanking active TSS” (state 2) of normal cells to “Bivalent/poised TSS” (state 16) of tumor cells. (**D**) GO and KEGG enrichment analysis of genes whose chromatin state transferred from “Flanking active TSS down” (state 3) of normal cells to “Bivalent/poised TSS” (state 16) of tumor cells.


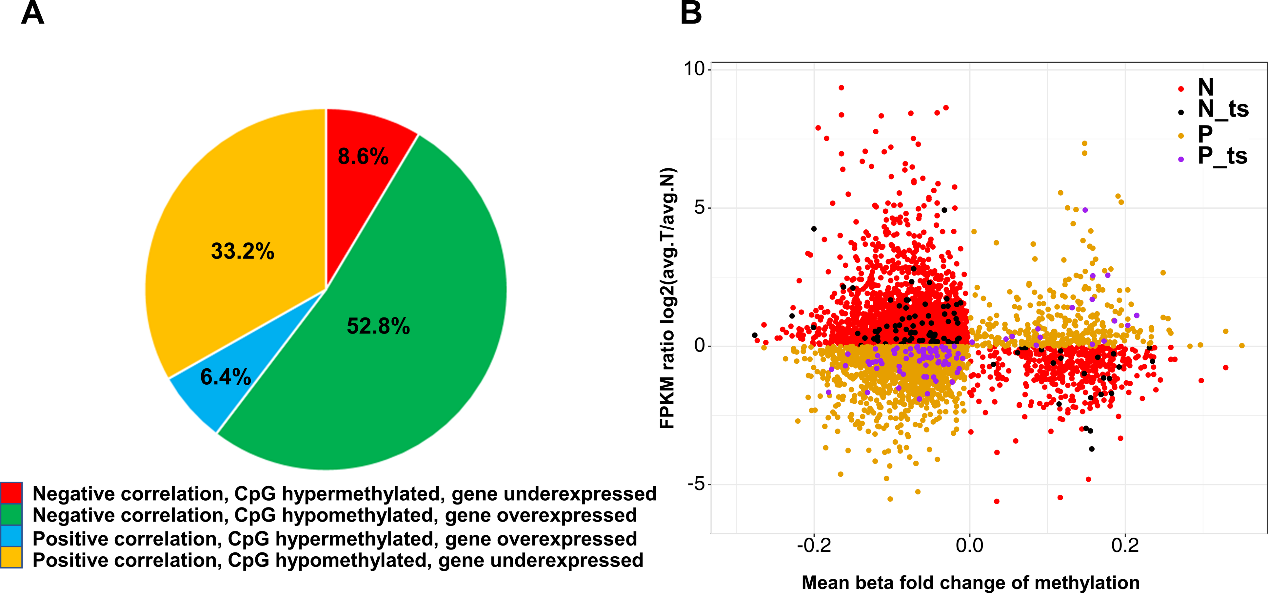


**Figure S4. Methylation-expression correlation of gastric cancer. (A)** Percentages of significantly correlated CpG sites. “underexpressed” and “overexpressed” referred to gene expression. **(B)** Correlation analyses were performed by calculating Spearman’s correlation coefficients (*P* values<0.05) for the ‘Negative’ and ‘Positive’ correlation group. The X-axis denotes the differential methylation between tumor and normal samples, and Y-axis denotes the differential gene expression between tumor and normal samples. ‘N’ and ‘P’ stand for the negative and positive correlation genes. ‘N_ts’ and ‘P_ts’ stand for the negative and positive correlation tumor suppressor genes.


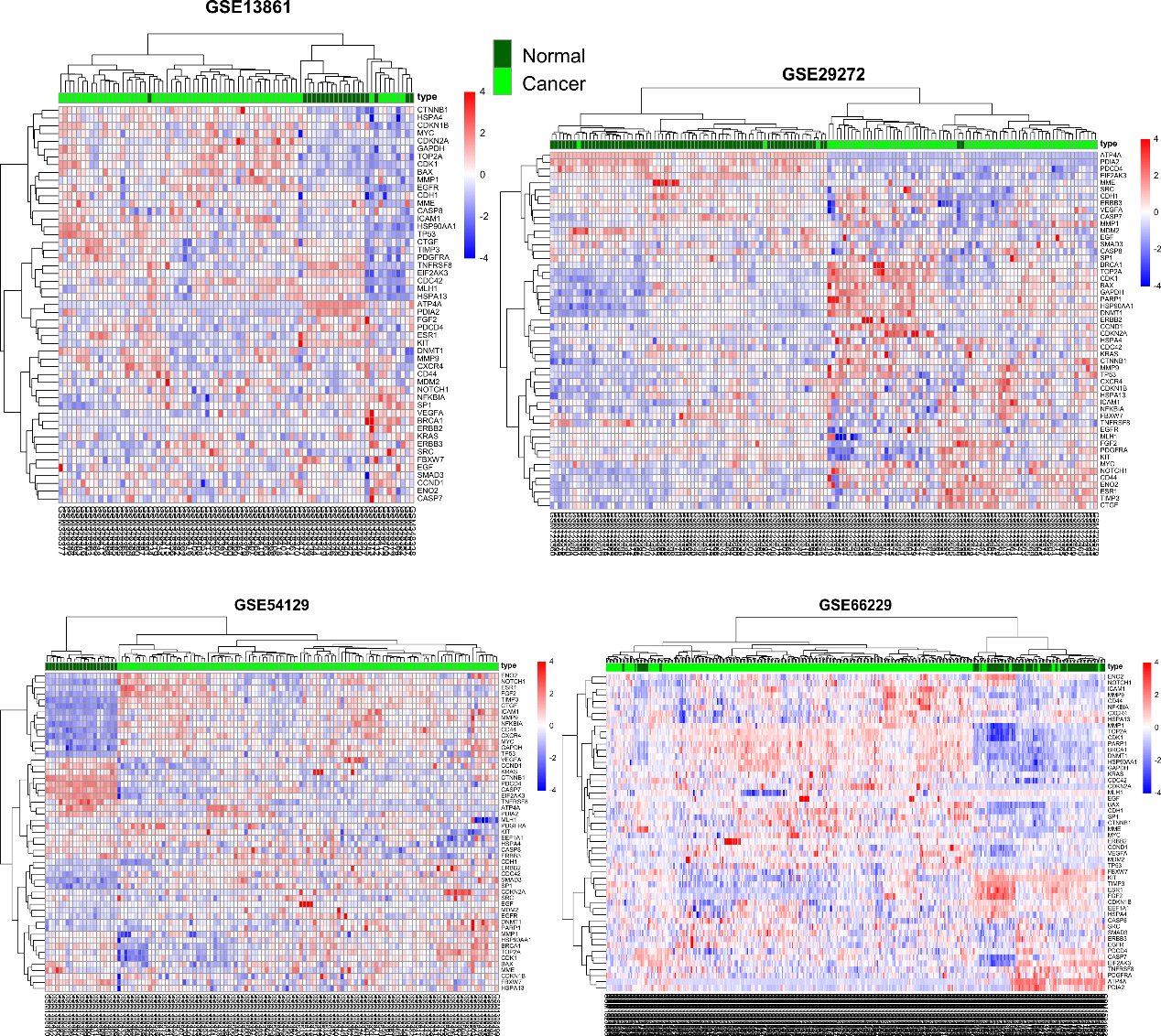


**Figure S5. Hierarchical clustering of independent gastric cancer samples cohorts.** Hierarchical clustering of four independent gastric cancer samples cohorts using expression profiles of 53 epigenetically regulated key genes. The independent cohorts followed by GSE13861, GSE29272, GSE54129, GSE66229 from GEO database


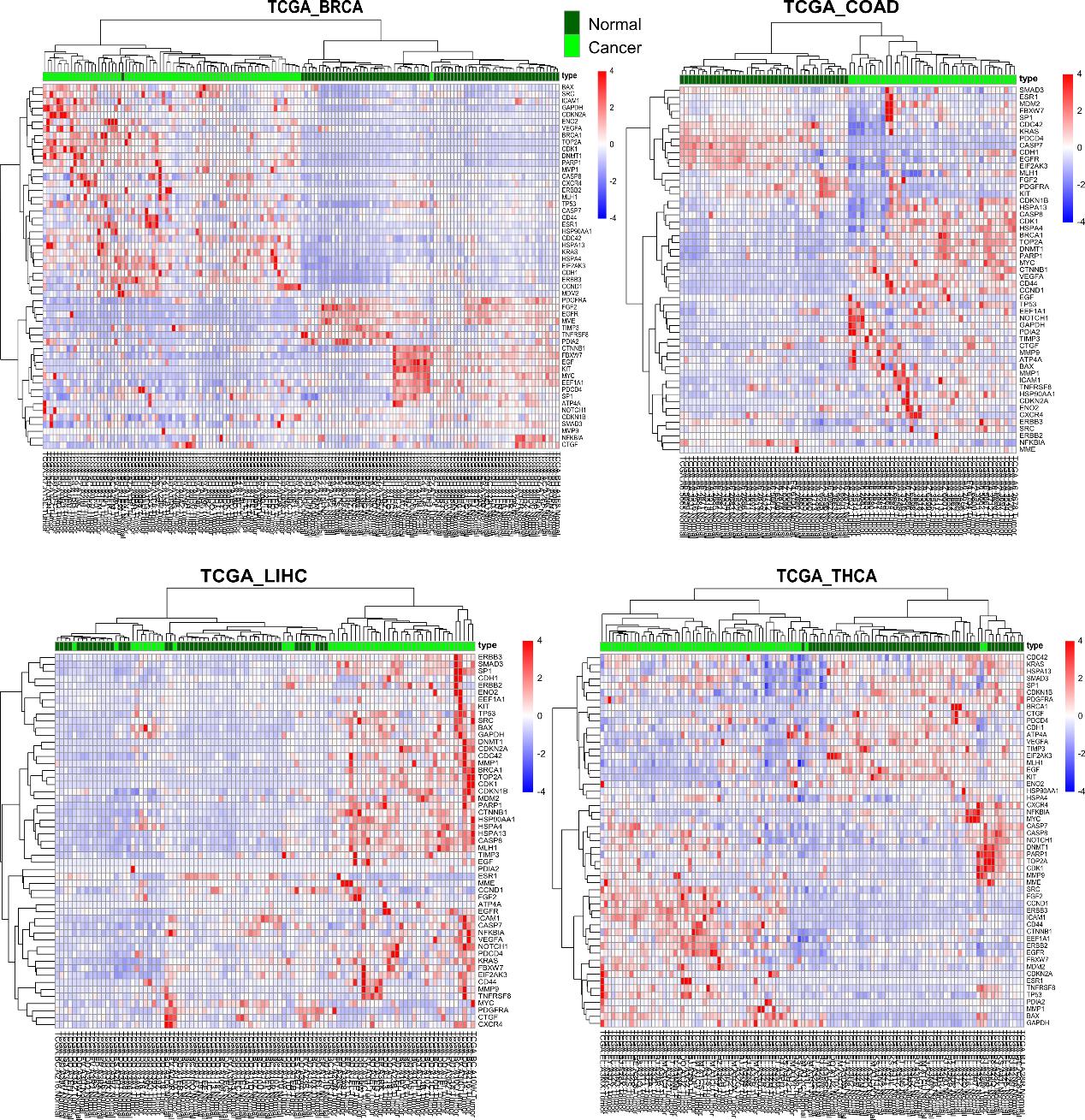


**Figure S6. Hierarchical clustering of four other different types of cancer.** Hierarchical clustering by using expressing profiles of epigenetically regulated key genes for breast invasive carcinoma (BRCA), colon adenocarcinoma (COAD), liver hepatocellular carcinoma (LIHC) and thyroid cancer (THCA) of TCGA database respectively.

**Table S1: Datasets used in this study.** All epigenetic modification data and gene expression data were collected from the primary sample research of the same patient cohort, including 19 primary GCs and 19 matched normal gastric tissues. ﻿Using high throughput sequencing, the primary sample research generated chromatin profiles covering multiple histone modifications, DNA methylation and gene expression.

| Data types |  | GEO accession |
| --- | --- | --- |
| ChIP-Seq | H3K4me1 | GSE51776 |
|  | H3K4me3 |  |
|  | H3K27ac |  |
|  | H3K27me3 |  |
|  | H3K36me3 |  |
| ﻿DNA methylation | - | GSE85464 |
| RNA-Seq | - | GSE85465 |

DNA methylation: Illumina HumanMethylation450 BeadChip
